# Occupational noise exposure is associated with hypertension in China: Results from project ELEFANT

**DOI:** 10.1371/journal.pone.0209041

**Published:** 2018-12-31

**Authors:** Akin Cayir, Timothy M. Barrow, Hao Wang, Hongbin Liu, Changping Li, Ning Ding, Yan Li, Choong-Min Kang, Liqiong Guo, Peng-hui Li, Hyang-Min Byun

**Affiliations:** 1 Vocational Health College, Canakkale Onsekiz Mart University, Canakkale, Turkey; 2 Human Nutrition Research Centre, Institute of Cellular Medicine, Newcastle University, Newcastle upon Tyne, United Kingdom; 3 Faculty of Health Sciences and Wellbeing, University of Sunderland, Sunderland, United Kingdom; 4 Tianjin Research Institute for Family Planning, Tianjin, China; 5 Department of Epidemiology and Statistics, School of Public Health, Tianjin Medical University, Tianjin, China; 6 School of Public Health, Tianjin Medical University, Tianjin, China; 7 Department of Environmental Health, School of Public Health, Harvard University, Boston, United States of America; 8 Department of Occupational & Environmental Health, School of Public Health, Tianjin Medical University, Tianjin, China; 9 School of Environmental Science and Safety Engineering, Tianjin University of Technology, Tianjin, China; Shanghai Diabetes Institute, CHINA

## Abstract

**Objectives:**

We investigated the association between occupational noise exposure and the risk of elevated blood pressure and hypertension by stage in young adults.

**Methods:**

We utilized 124,286 young adults (18–40 years) from the Project ELEFANT study. We categorized occupational noise exposure as *high* (75 dBA noise exposure for more than 4 hours per day) or *low*, and measured blood pressure (mmHg) and categorized participants by hypertension stage (normal, elevated, Stage 1, Stage 2). We applied adjusted logistic regression models to identify associations with hypertension risk, and we further examined the noise-BMI, noise-gender, and noise-residence interactions on hypertension risk in separate models.

**Results:**

High occupational noise exposure was associated with increases in blood pressure among participants with elevated blood pressure (Estimate = 0.23, 95% CI: 1.09, 1.46, *p* = 0.0009), in Stage 1 hypertension (Estimate = 0.15, 95% CI: 1.06, 1.25, *p* = 0.0008), and in Stage 2 hypertension (Estimate = 0.41 95% CI: 1.31, 1.73, *p<*0.0001). Likewise, noise exposure-BMI interaction was consistently positively associated with increases in blood pressure in participants with elevated blood pressure (Estimate = 0.71, 95% CI: 1.55, 2.69, *p*<0.0001), in Stage 1 hypertension (Estimate = 0.78, 95% CI: 1.82, 2.61, *p*<0.0001), and in Stage 2 hypertension (Estimate = 2.06, 95% CI: 5.64, 10.81, *p*<0.0001). The noise exposure-male interaction showed higher risk for hypertension compared to the noise exposure-female interaction in participants with elevated blood pressure (Estimate = 1.24, 95% CI: 2.56, 4.71, *p*<0.0001), Stage 1 (Estimate = 1.67, 95% CI: 4.34, 6.42, *p*<0.0001) and Stage 2 hypertension (Estimate = 1.70, 95% CI: 3.86, 7.77, *p*<0.0001). Finally, we found that noise exposure-urban interaction was consistently associated with an increase in blood pressure in elevated blood pressure (Estimate = 0.32, 95% CI: 1.19, 1.62, *p*<0.0001) and in Stage 2 hypertension (Estimate = 0.44, 95% CI: 1.31, 1.80, *p*<0.0001).

## Introduction

Noise is a highly prevalent environmental exposure in the United States [1], in European countries [2, 3] and in Asian countries [4, 5]. The World Health Organization (WHO) has classified noise pollution as the second leading environmental cause of health problems, after particulate matter air pollution [6]. Living and working conditions affect noise exposure by its intensity, the complexity of sounds, and frequency [7], with transport and industrial activities the leading sources of exposure. There is increasing evidence for a negative impact of noise exposure upon human health [8], including both auditory as well as non-auditory health endpoints such as annoyance, sleep disturbance, reading impairment in children, hypertension and cardiovascular diseases (CVD), including arteriosclerosis, ischemic heart disease, and stroke [9]. The WHO has estimated that the number of healthy life years lost annually due to noise exposure is 61,000 for CVD, 45,000 for cognitive impairment of children, 903,000 for sleep disturbance, 22,000 for tinnitus, and 654,000 for annoyance in western European countries [10].

According to the WHO, ischemic heart disease, stroke and hypertensive heart diseases were the leading causes of death in 2015 [11]. In this context, the role of noise exposure as contributor to CVD becomes a crucial concern of public health. Indeed, environmental noise exposure has been implicated in increased diagnosis of hypertension, hospital admission and premature mortality [2]. However, while a number of studies have reported hypertension and/or blood pressure as being positively associated with noise exposure [12–15], others have reported negative associations [16, 17]. These contrasting findings may be due to the inherent complexity in studying the effect of noise exposure and underlines the need for further research into its effects upon human health. Furthermore, there is a need to better understand how the effect of noise pollution exposure may be mediated by other factors, such as lifestyle and genetics.

In this study, we investigated the effect of chronic occupational noise exposure upon the development of hypertension in young adults. We utilized 124,286 individuals (18–40 years) from within Project Environmental and LifEstyle Factors iN metabolic health throughout life-course Trajectories (ELEFANT) to estimate risk of hypertension by stage (elevated blood pressure, Stage 1, and Stage 2) with noise exposure, and examined the interaction of noise-BMI, noise-gender, and noise-residence on hypertension risk in separate models. We report that noise exposure is associated with increased blood pressure in elevated blood pressure, Stage 1 and Stage 2, and displayed interaction with BMI, gender and residence.

## Methods

### Study design and participants

Project ELEFANT is a newly developed population study based in Tianjin, China that comprises three population studies: Baby ELEFANT (age 0, *n* = 48,762); Young ELEFANT (mean age 30, *n* = 366,474); and Elderly ELEFANT (mean age 65, *n* = 6,503) [18]. Complete blood counts, blood pressure, fasting blood glucose level, serum alanine amino transferase enzyme level, high sensitivity C-reactive protein (hs-CRP) serum level, thyroid stimulating hormone (THS) level, body mass index (BMI) and other clinical characteristics were measured during routine check-ups. The participants also completed questionnaires between 2011 and 2017 which included sections on demographics, familial history of disease, medication uses and dietary intakes. We utilized the Young ELEFANT participants who were enrolled in 2014 and 2015, aged between 18 and 40 and with an equal distribution between the two genders (*n* = 124,286). The protocol of this study was approved by the Institutional Review Board of the Tianjin Medical University, and participants gave written informed consent prior to participation in the study.

### Blood pressure measurement and classification of hypertension

Systolic blood pressure (SBP, mmHg) and diastolic blood pressure (DBP, mmHg) were measured according to criterion of the JNC 7 (Seventh Report of the Joint National Committee on Prevention, Detection, Evaluation, and Treatment of High Blood Pressure) [19]. Blood pressure (BP) was measured twice in the upper left arm after 5–10 min of rest in a seated position using an automated device (HBP-9021J, Omron, Japan). The mean of these two measurements was used for further analysis. Participants were classified as normal, elevated blood pressure, Stage 1, and Stage 2 hypertension using SBP and DBP values according to the ACC/AHA Clinical Practice Guidelines published in 2017 [20].

### Assessment of variables

To assess the occupational noise exposure of the participants, we constructed questionnaires to independently capture the occupation of the individual and their level of noise exposure (high/low). High noise exposure was defined as exposure to levels higher than 75 dBA for more than four hours per day. The participants reported more than 100 different types of occupation, which were categorized into three groups based upon The International Standard Classification of Occupations (ISCO) [21]. In our population, there was no correlation between occupation and occupational noise exposure, demonstrating a high level of variance even within similar occupations. We therefore categorized to three occupational groups for our analysis: 1) skilled agricultural workers; 2) elementary occupations, technicians and associate professionals, service and sales workers; and 3) professionals and clerical support workers.

We followed Chinese guidelines to define BMI categories, with BMI below 24 defined as normal weight, between 24 and 28 as overweight, and higher than 28 as obese [22].

### Statistical analysis

The relationship between noise exposure and blood pressure as continuous variables was investigated using multivariate regression analysis. The relationships between each blood pressure classification (elevated, Stage 1, and Stage 2 hypertension) with noise exposure were evaluated using logistic regression analysis. In both the logistic and regression models, we adjusted the model with the following covariates: age (continuous); BMI (continuous); gender (male/female); occupation (3 categories); residence (urban/rural); education (basic/higher); smoking (yes/no); number of cigarettes per day; drinking (yes/no); and noise exposure (high/low). We fitted a logistic regression model to investigate all interaction and all the models were adjusted with the same variables.

Log(p/1−p)=β0+β1Noise+β2x1+…+βn+1xn (1)

Where x1… xn are the covariates, Noise is the noise exposure, and *p* is the probability of having one of the stages of the hypertension. All analyses were performed using SAS 9.4 (SAS Institute, Cary, NC).

## Results

We analyzed 118,143 participants who were aged between 18 and 40, and who had complete data for all covariates of the adjusted model. The basic characteristics of participants are shown in Table 1. The mean age was 29.64, and there were a similar number of men and women. In the population, 3,016 individuals were exposed to high levels of occupational noise (>75 dBA for more than four hours per day), while 115,127 individuals did not. Blood pressure levels were normal for 43.8% of the cohort, 7.3% had elevated blood pressure, 42.9% had Stage 1 hypertension, and 6.1% had Stage 2 hypertension.

**Table 1:** Baseline characteristics of the cohort.

Characteristics	Occupational noise exposure Low *n* = 115,127	Occupational noise exposure High *n* = 3,016	Total *n* = 118,143
Age (y)	29.63±4.14	30.03±3.67	29.64±4.13
BMI (kg/m^2^)	23.70±4.49	24.62±5.07	23.72±4.51
Gender, *n* (%)			
Men	56,464 (96.32)	2,158 (3.68)	58,662
Women	58,663 (98.56)	858 (1.44)	59,521
Occupations*, *n* (%)			
1	66,960 (99.23)	520 (0.77)	67,480
2	18,780 (92.56)	1,510 (7.44)	20,290
3	29,387 (96.75)	986 (3.25)	30,377
Residence, *n* (%)			
Urban	39,819 (94.93)	2,127 (5.07)	41,946
Rural	75,308 (98.83)	889 (1.17)	76,197
Education, *n* (%)			
Basic	67,067 (98.27)	1,180 (1.73)	68,247
Higher	48,060 (96.32)	1,836 (3.68)	49,896
Smoking Habit, *n* (%)			
No	98,349 (98.13)	1,873 (1.87)	100,222
Yes	16,778 (93.62)	1,143 (6.38)	17,921
Number of cigarettes per day	1.54±4.49	4.27±10.96	1.61±4.78
Drinking, *n* (%)			
No	94,113 (98.66)	1,279 (1.34)	95,392
Yes	21,014 (92.37)	1,737 (7.63)	22,751
Blood Pressure Classifications, *n* (%)			
Normal	50,718 (98.09)	985 (1.91)	51,703
Elevated	8,277 (96.68)	284 (3.32)	8,561
Stage 1	49,346 (97.26)	1,389 (2.74)	50,735
Stage 2	6,786 (94.99)	358 (5.01)	7,144

*1) skilled agricultural workers, 2) elementary occupations, technicians and associate professionals, service and sales workers, 3) professionals and clerical support workers.

Blood pressure levels were higher among participants with high occupational noise exposure, and this was consistent within each of classification of hypertension/elevated blood pressure (Table 2). The mean SBP among all participants with high occupational noise exposure was 119.39 mmHg in comparison to 115.67 mmHg in those without (*p*<0.0001, *t-test*). The mean DBP of participants with high noise exposure levels (77.07 mmHg) was significantly higher than those with low levels of exposure (74.91 mmHg) (*p*<0.0001, *t-test*). SBP and DBP were both significantly associated with noise exposure in the adjusted model (*p*<0.0001 and *p* = 0.004, respectively). In the multivariate linear regression model, SBP (β = 0.02, 95% CI: 0.008, 0.02) and DBP (β = 0.008, 95% CI: 0.002, 0.01) were increased with high exposure (Table 3).

**Table 2:** Descriptive statistics of SBP and DBP by noise exposure.

	SBP			DBP		
	Occupational noise exposure Low	Occupational noise exposure High	p-Value*	Occupational noise exposure Low	Occupational noise exposure High	p-Value*
Total	115.66±10.84	119.39±12.22	<0.0001	74.91±8.54	77.07±9.03	<0.0001
Normal	106.82±6.04	107.42±6.58	<0.0001	67.82±5.20	68.33±5.15	<0.0001
Elevated	121.27±2.38	122.56±2.97	<0.0001	71.65±4.06	71.43±4.66	<0.0001
Stage 1	120.68±5.24	122.19±6.84	<0.0001	80.34±2.04	80.61±3.19	<0.0001
Stage 2	138.48±13.78	139.02±11.52	<0.0001	92.41±8.70	91.84±7.51	<0.0001

SBP: Systolic Blood Pressure, DPB: Diastolic Blood Pressure

* t-test

**Table 3:** Association between blood pressure and occupational noise exposure.

Dependent Variable	Model	B	β	*p-Value*	Adjusted R^2^ Model	95% CI
SBP	Noise High	0.01	0.02	0.000	0.19	0.008, 0.02
DBP	Noise High	0.006	0.008	0.004	0.18	0.002, 0.01

SBP: Systolic Blood Pressure, DPB: Diastolic Blood Pressure B: Unstandardized Coefficient, β: Standardized Coefficient Predictors: (Constant), noise exposure, BMI, age, gender, occupation, residence, education, smoking habit, number of cigarettes per day, and drinking habit.

The odds ratio (OR) of elevated blood pressure with high occupational noise exposure was 1.77 (95% CI: 1.55, 2.02), 1.45 (95% CI: 1.33, 1.57) for Stage 1 hypertension, and 2.72 (95% CI: 2.40, 3.07) for Stage 2 hypertension (Table 4). In this logistic regression analysis, occupational noise exposure was associated with increases in blood pressure among participants with elevated blood pressure (Estimate = 0.23, 95% CI: 1.09, 1.46, *p* = 0.0009), in Stage 1 hypertension (Estimate = 0.15, 95% CI: 1.06, 1.25, *p* = 0.0008), and in Stage 2 hypertension (Estimate = 0.41 95% CI: 1.31, 1.73) (Table 5). The ORs for the risk of elevated blood pressure, Stage 1 and Stage 2 hypertension with high occupational noise exposure were 1.26, 1.16, and 1.51, respectively (Table 5).

**Table 4:** Odds ratios for hypertension and noise exposure.

	Noise (n)	OR	95% CI	*p-Value* *
**Elevated**	Low (8277)	Reference	-	-
	High (284)	1.77	1.55, 2.02	<0.0001*
**Stage 1**	Low (49346)	Reference	-	-
	High (1389)	1.45	1.33, 1.57	<0.0001*
**Stage 2**	Low (6786)	Reference	-	-
	High (358)	2.72	2.40, 3.07	<0.0001*

* Fisher’s Exact Test, OR: Odds Ratio

**Table 5:** Logistic model for associations between noise exposure and hypertension.

	Noise Exposure (*n*)	Estimate	ORs†	95% CI	*p-Value*
Elevated	Low (8277)	Reference		-	-
	High (284)	0.23	1.26	1.09, 1.46	0.0009*
Stage 1	Low (49346)	Reference		-	-
	High (1389)	0.15	1.16	1.06, 1.25	0.0008**
Stage 2	Low (6786)	Reference		-	-
	High (358)	0.41	1.51	1.31, 1.73	1.04E-08*

*Logistic model adjusted for BMI, age, gender, occupation, residence, education, smoking habit, number of cigarettes per day, and drinking habit.

** Logistic model adjusted for BMI, age, occupation, residence, education, smoking habit, and number of cigarettes per day.

^†^ The odds ratio associated with noise exposure.

We investigated the interaction effect of noise exposure on blood pressure in logistic models. We categorized individuals as under/healthy weight (BMI <24) or overweight/obese (BMI≥24). The noise exposure-BMI interaction was consistently positively associated with an increase in blood pressure in participants with elevated blood pressure (Estimate = 0.71, 95% CI: 1.55, 2.69, *p*<0.0001), Stage 1 hypertension (Estimate = 0.78, 95% CI: 1.82, 2.61, *p*<0.0001), and Stage 2 hypertension (Estimate = 2.06, 95% CI: 5.64, 10.81, *p*<0.0001) (Table 6). Thus, the interaction was associated with ORs of 2.03, 2.18 and 7.84 for elevated blood pressure, Stage 1 and Stage 2 hypertension, respectively (Table 6). Analysis of the interaction of noise exposure with gender on blood pressure revealed that noise exposure-male interaction showed higher risk for hypertension compared with the noise exposure-female interaction for elevated blood pressure (Estimate = 1.24, 95% CI: 2.56, 4.71, *p*<0.0001), Stage 1 (Estimate = 1.67, 95% CI: 4.34, 6.42) and Stage 2 hypertension (Estimate = 1.70, 95% CI: 3.86, 7.77) (Table 6). The noise exposure-gender interaction was associated with ORs of 5.31 and 5.47 for the risk of Stage 1 and Stage 2 hypertension respectively (Table 6). Finally, analysis by residence revealed that the noise exposure-urban interaction was significantly associated with increases in blood pressure in elevated blood pressure (Estimate = 0.32, 95% CI: 1.19, 1.62, *p*<0.0001) and Stage 2 hypertension (Estimate = 0.44, 95% CI: 1.31, 1.80, *p*<0.0001), with odds ratios of 1.38 and 1.55 respectively (Table 6).

**Table 6:** Logistic model for association between noise exposure-BMI, -gender, and -residence interaction and blood pressure.

			Estimate	ORs†	95% CI	*p-Value*
Noise Exposure*BMI**		Noise Low*BMI	Reference		-	-
	Elevated	Noise High*BMI	0.71	2.03	1.55, 2.69	<0.0001*
	Stage 1	Noise High*BMI	0.78	2.18	1.82, 2.61	<0.0001*
	Stage 2	Noise High*BMI	2.06	7.84	5.64, 10.81	<0.0001*
Noise Exposure*Gender		Noise High*Female	Reference		-	-
	Elevated	Noise High*Male	1.24	3.54	2.56, 4.71	<0.0001*
	Stage 1	Noise High*Male	1.67	5.31	4.34, 6.42	<0.0001*
	Stage 2	Noise High*Male	1.70	5.47	3.86, 7.77	<0.0001*
Noise Exposure*Residence		Noise High*Rural	Reference		-	-
	Elevated	Noise High*Urban	0.32	1.38	1.19, 1.62	<0.0001*
	Stage 1	Noise High*Urban	-0.03	-	0.86–1.07	0.57
	Stage 2	Noise High*Urban	0.44	1.55	1.31, 1.80	<0.0001*

*Logistic model adjusted for age, gender, occupation, residence, education, smoking habit, number of cigarettes per day, and drinking habit.

**We created a dichotomized variable for BMI as 24 kg/m^2^ was chosen as the threshold.

^†^ The odds ratio associated with noise exposure.

## Discussion

In this study, we investigated the association between occupational noise exposure and hypertension by stage in young adults from the Project ELEFANT study. Additionally, we analyzed the interaction of BMI, gender and residence with noise exposure on blood pressure. Our results revealed a consistent pattern of increased risk of hypertension by stage (elevated blood pressure, Stage 1 and Stage 2) and higher blood pressure among individuals with high levels of occupational noise exposure, with greater effect in overweight/obese individuals and men.

While the relationship between noise exposure and hypertension and/or blood pressure has been extensively investigated, the results to date have been inconsistent. A meta-analysis of 43 studies reported that 5 dB(A) of occupational and traffic noise exposure were associated with a relative risk of hypertension of 1.14 and 1.26, respectively [23]. Recent studies have provided evidence in support of these findings, with higher SBP and DBP levels in individuals exposed to occupational noise and significantly higher risk of hypertension [4, 12]. This is further supported by studies reporting aircraft and traffic noise exposure as associated with increased SBP and DBP, and relative risks of hypertension of 1.14 and 1.59 respectively [3]. However, other studies have reported no increased risk of the hypertension with occupational noise exposure [16] or even a negative association [24], and no effect upon DBP [13]. These inconsistencies may in part be the product of the difficulty in defining noise exposure. Health outcomes depend greatly on the context and characteristics of acoustic insult; not only the intensity, but also stimulus duration, frequency content, and predictability [8]. However, some of the inconsistency may also be the product of small cohort sizes, inappropriate measures of exposure time and level, and an inability to adjust for potential confounding factors. In our study, we were able to adjust for a range of confounding factors such as age, gender, BMI, smoking, and alcohol consumption. Furthermore, to the best of our knowledge, our study is the first to demonstrate that noise exposure is associated with greater physiological effects and risk of hypertension amongst males, individuals who are overweight or obese, and those in urban locations. Our findings may aid in identifying at-risk individuals who would most benefit from therapeutic intervention.

While occupational noise has been reported to be associated with hypertension, and other forms of heart disease, the mechanisms underpinning these associations have not yet been elucidated. However, evidence from the study of noise exposure upon blood pressure may provide insight into these processes. Acute noise exposure has effects on the endocrine and sympathetic nervous systems that result in physiological changes such as increased heart rate, blood pressure, vasoconstriction, production of stress hormones [25]. Noise may induce the production of stress factors via both direct and indirect pathways leading to changes in blood glucose, pressure, and lipid concentration levels [26], and chronic noise exposure is associated with alterations in a number of biochemical molecules implicated in CVD [27].

Epigenetic mechanisms frequently mediate the effects of environmental exposures in relation to health and disease. Alterations in DNA methylation have been implicated with many diseases including cancer and CVD. Importantly, several studies have demonstrated that epigenetic changes in response to different stresses including disruption of circadian system, chronic social stress, and famine stress from the Dutch Hunger [28]. In particular, changes in the epigenetic regulation of the glucocorticoid receptor gene *NR3C1* has been reported in response to different forms of stress [29, 30]. In our previous work, we have demonstrated global and gene-specific (*Bdnf*, *Comt*, *Mc2r*) DNA methylation changes in the brain response to both short-term and long-term noise exposure [31]. These epigenetic changes were significantly associated with changes in body weight and blood pressure, and we therefore speculate that epigenetic factors may mediate the risk of elevated blood pressure and hypertension in individuals with high levels of occupational noise exposure. This is supported by the wealth of evidence for the role of epigenetic alterations in biological process linked with hypertension, atherosclerosis, and inflammation [32].

There are several limitations in our study. Firstly, more precise measurement of noise exposure would potentially increase the power and accuracy of analysis, but this would have been prohibitively expensive in a large cross-sectional study such as our own. It is also known that noise exposures are correlated with air pollution exposure, such as via traffic. In this study, we were unable to account for ambient and traffic air pollution, although we were able to adjust for residential area and occupation. Finally, due to the lack of standard and insightful measures of noise exposure (e.g. intensity, stimulus duration, frequency content, predictability), our study has suffered from the same inherent difficulty as others in describing exposure levels. However, it should also be noted that our study has several key strengths. Most notably, we have been able to leverage a very large cohort of young adults who are otherwise healthy and provide sufficient statistical power to accurately analyse the impact of exposure upon blood pressure and risk of hypertension. Furthermore, due to the well-characterized nature of the cohort, we were able to adjust for a large number of potential confounders to more precisely study the effect of occupational noise exposure.

In summary, our study has revealed that occupational noise exposure is associated with higher risk of elevated blood pressure and hypertension in young adults, and we have demonstrated an interaction of noise with gender and BMI. Further work is required to investigate the biological mechanisms that may explain these associations.
